# Effects of FM0807, a novel curcumin derivative, on lipopolysaccharide-induced inflammatory factor release via the ROS/JNK/p53 pathway in RAW264.7 cells

**DOI:** 10.1042/BSR20180849

**Published:** 2018-10-17

**Authors:** Yilong Wu, Zhiwei Liu, Weifang Wu, Su Lin, Nanwen Zhang, Honglin Wang, Shuangyu Tan, Peimin Lin, Xiaole Chen, Lixian Wu, Jianhua Xu

**Affiliations:** 1 The Graduate School of Fujian Medical University, Fuzhou, P.R. China; 2 The School of Clinical Medicine,Fujian Medical University, Fuzhou, P.R.China; 3 Department of Anesthesiology,Fuzhou Children`s Hospital of Fujian Province, Fuzhou, P.R.China; 4 Liver Research Center,the First Affiliated Hospital of Fujian Medical University, Fuzhou, P.R.China; 5 The School of Pharmacy,Fujian Medical University, Fuzhou, P.R. China; 6 ThKey Laboratory of Natural Medicine Pharmacology in Fujian Province, Fuzhou, P.R.China

**Keywords:** Curcumin, inflammatory cytokine, LPS, RAW 264.7, sepsis

## Abstract

**Purpose:** Sepsis is a systemic inflammatory response caused by infection. Curcumin is known to have antioxidant and anti-inflammatory activities. FM0807, a curcumin derivative, was investigated in the present study to determine its effect on cytokines and the possible molecular mechanism. **Main methods:** The experiments were carried out in lipopolysaccharide (LPS)-induced RAW 264.7 cells. Cell viability was measured by MTT assay. ELISA, Griess assays, fluorescence-based quantitative PCR, flow cytometric analysis, 2′,7′-dichlorodihydrofluorescein diacetate (DCFH-DA) experiments, and Western blotting were carried out to assess the potential effects of FM0807 on LPS-induced RAW 264.7 cells. **Significant findings:** FM0807 had no cytotoxic effects on RAW 264.7 cells. Furthermore, pretreatment with FM0807 inhibited the inflammatory factor tumor necrosis factor-α (TNF-α), interleukin (IL) 1β (IL-1β), IL-6, and inducible nitric oxide synthase (iNOS) at the protein and gene levels. FM0807 also inhibited the production of reactive oxygen species (ROS) and apoptosis. In addition, the activation of the ROS/JNK (c-jun NH_2_-terminal kinase)/p53 signaling pathway was inhibited by FM0807 in RAW 264.7 cells *in vitro*. **Conclusion:** FM0807 has anti-inflammatory activity *in vitro*, which suggests a potential clinical application in sepsis. The anti-inflammatory activity of FM0807 may be mediated by the ROS/JNK/p53 signaling pathway.

## Introduction

Sepsis is a systemic inflammatory response caused by a dysregulated host response to infection. Sepsis is one of the most serious and common causes of death in the intensive care unit (ICU). A meta-analysis reported that the hospital mortality was 17% for sepsis and 26% for severe sepsis from 1979 to 2015 in seven high-income countries. And a tentative extrapolation from high-income country data suggests global estimates of 31.5 million sepsis and 19.4 million severe sepsis cases, with potentially 5.3 million deaths annually [1]. The pathogenesis of sepsis is not very clear. Sepsis becomes to be a huge therapeutic challenge. In recent years, studies have shown that oxidative stress may be one of the factors in the cause of sepsis [2–4]. Under normal physiological conditions, the formation and clearance of reactive oxygen species (ROS) and reactive nitrogen species (RNS) are in dynamic state, ROS and RNS are pivotal to the defense against invading pathogens during sepsis by performing intracellular signaling for several cytokines and growth factors, participating in intracellular killing of bacterial pathogens in phagocytosis [5]. In infection, poisoning, trauma, and other pathological conditions, the body has a variety of ways to produce ROS and RNS. The oxidative stress and nitrosative stress come, which are caused by overwhelming production of ROS and RNS. ROS play a wide range of capabilities due to action in many ways and numerous places simultaneously [6]. ROS can directly play a toxic effect on the cells. In the process of oxidative stress, ROS can cause protein and nucleic acid to be oxidized, and can lead to mitochondrial membrane destruction, then the cells apoptosis. Caspases such as caspase-3 and caspase-9 play a vital role in apoptosis. Many studies showed that ROS can lead to activation of the mitogen-activated protein kinase (MAPK) signaling pathway [7,8]. There are three main MAPKs, including c-jun NH_2_-terminal kinases (JNK), extracellular signal-regulated kinases (ERKs), and p38 [9,10]. p53 is a potent inducer of apoptosis. Recently, many studies suggested that the ROS/JNK/p53 pathway plays a vital role in apoptosis [11,12]. ROS activates the MAPK signaling pathway to induce apoptosis and the production of a large number of inflammatory cytokines [13–15].

**Figure 1:**
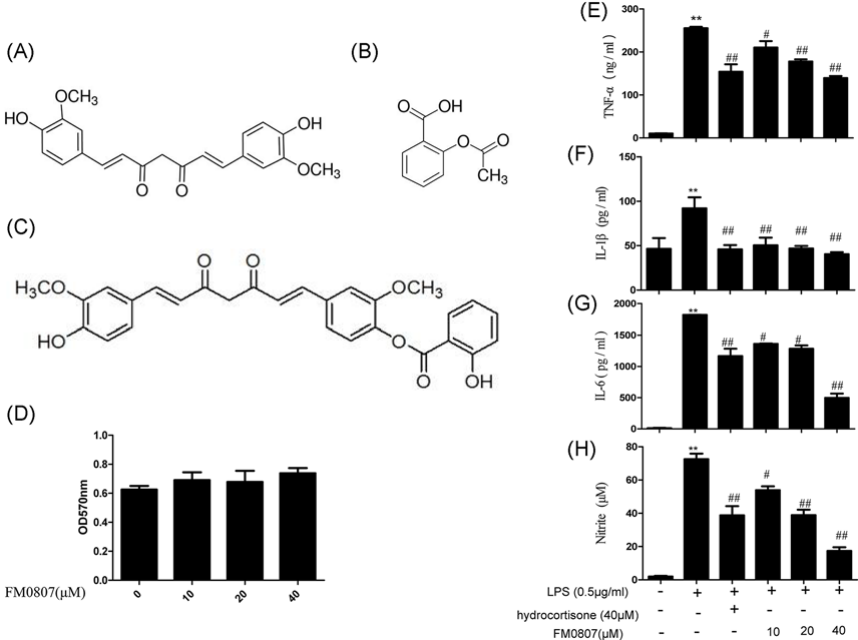
Chemical structures of Cur, aspirin, and FM0807 and inhibitory effects of FM0807 on LPS-induced cytokines (**A**) Chemical structure of Curcumin; (**B**) chemical structure of aspirin; (**C**) chemical structure of curcumin analog FM0807. Molecular formula: C_28_H_24_O_8_, molecular weight: 488.15 g.mol^−1^; (**D**) viability of RAW 264.7 after treatment of different concentrations of FM0807. At a given concentration, FM0807, had no significant effect on the cellular activity of RAW 264.7 compared with the control group; (**E**) the release of TNF-α; (**F**) the release of IL-1β; (**G**) the release of IL-6; (**H**) the release of nitrite. Values were mean ± S.D. (*n*=3); **P<0.01 compared with the control group; ^#^*P*<0.01 and ^##^*P*<0.05 compared with the LPS-treated group. Abbreviations: IL, interleukin, LPS, lipopolysaccharide, TNF-α, tumor necrosis factor-α.

Curcumin has a wide range of pharmacological effects, such as antitumor, hypolipidemic, and anti-inflammatory effects [16–18]. However, its poor water-solubility and low bioavailability restrict its use. To address this problem, a number of derivatives and analogs of Cur have been developed in our lab, such as FM0807 (2-hydroxy-4-[(1E, 6E)-7-(4-hydroxy-3-methoxyphenyl)-3,5-dioxo-1,6-heptadien-1-yl]-2-methoxyphenyl ester), which incorporated a 2-hydroxy-benzoic acid chain into Cur that retains the β-diketone structure and exhibits better anti-inflammatory activity than that of Cur [19]. FM0807 can also be considered as the combined compound of Cur and aspirin (the structures of Cur, aspirin, and FM0807 are shown in Figure 1A–C). In the present study, a curcumin derivative, FM0807, (Figure 1C) was synthesized, and its anti-inflammatory activities were evaluated *in* RAW264.7 cells. To explore the molecular mechanisms underlying these effects, we also investigated the possible involvement of the ROS/JNK/p53 signaling pathway.

## Materials and methods

### Chemicals and reagents

The curcumin derivative FM0807 was synthesized by Clinical Pharmacology Research Center of Fujian Medical University. Lipopolysaccharide (LPS) (*Escherichia coli* 0111: B4) and DMSO were obtained from Sigma–Aldrich (St. Louis, MO, U.S.A.), and the Annexin-V FITC Apoptosis Detection Kit was purchased from Becton, Dickinson and Company (San Jose, CA, U.S.A.). Dulbecco’s modified Eagle’s medium (DMEM) and low-endotoxin FBS were purchased from Gibco Company (Grand Island, NY, U.S.A.). ELISA kits for tumor necrosis factor-α (TNF-α), interleukin (IL) 1β (IL-1β), and IL-6 were obtained from Santa Cruz Biotechnology (Dallas, TX, U.S.A.). Primary antibodies against p-JNK1/2, p-ERK1/2, JNK1/2, ERK1/2, p53, caspase-9, caspase-3, and β-actin were purchased from Cell Signaling Technology (Danvers, MA, U.S.A.). PMSF was purchased from Roche Company (Switzerland). The nitric oxide (NO) assay kit, BCA protein content determination kit, and reactive oxygen test kit were obtained from Beyotime Institute of Biotechnology (Shanghai, China).

### Cell lines and sample treatment

The RAW 264.7 mouse macrophage cell line was purchased from Shanghai Institute of Cellular Biology of the Chinese Academy of Sciences (Shanghai, China). The cells were cultured in DMEM supplemented with 10% FBS, penicillin (100 U/ml), and streptomycin (100 U/ml) in a humidified incubator containing 5% CO_2_ at 37°C. When grown to 70–80% confluence, the cells were subjected to passaging. The medium was replaced every 1–2 days. The concentration of the cell suspension was adjusted to 1 × 10^5^/ml. Then, 1 ml samples of the suspension were added to 24-well plates and cultured in a humidified incubator containing 5% CO_2_ at 37°C. The cells were divided into six groups: the control group (the cells were untreated), the LPS group (the cells were treated with 0.5 μg/ml LPS), the positive control + LPS group (the cells were treated with 40 mM hydrocortisone plus 0.5 μg/ml LPS), and the FM0807+LPS groups (the cells were treated with 10, 20, or 40 μM FM0807 plus 0.5 μg/ml LPS). The RAW 264.7 cells were pretreated with different concentrations of FM0807 and hydrocortisone for 20 h before treatment with 0.5 μg/ml (final concentration) LPS for 4 h, with 200 µl of supernatant per well. Each group was represented by three wells, and the supernatants were preserved at −20°C.

### Cell viability assay

RAW 264.7 cells were seeded in 96-well plates at a density of 1 × 10^5^/ml in a volume of 200 µl/well. After incubation for 24 h at 37°C, the cells were treated with FM0807 at the indicated concentrations for 24 h, followed by the addition of 5 mg/ml MTT solution to each well, and the plates were further incubated for 4 h at 37°C. The supernatant was removed, and 150 µl of DMSO was added to each well to solubilize the water-insoluble purple formazan crystals. The absorbance at a wavelength of 570 nm was measured using a microplate reader (Bio-Rad Laboratories, Inc., Hercules, CA, U.S.A.). The percentage of viable cells was estimated and compared with that of untreated control cells.

### Measurement of NO

The Griess assay was performed to detect NO [20]. RAW 264.7 cells were seeded in 96-well plates at a density of 1 × 10^5^/ml and a volume of 200 µl/well. After incubation for 24 h at 37°C, the cells were treated with hydrocortisone and FM0807 at the indicated concentrations for 20 h in serum-free medium, prior to the addition of LPS (0.5 μg/ml). After 4 h, the culture supernatants were collected and reacted with Griess reagent (1% sulphanilamide/0.1% naphthyl ethylene diamine dihydrochloride/2% phosphoric acid) in a 96-well plate. Then, the optical density was detected at 540 nm with a microplate reader.

### Determination of TNF-α, IL-1β, and IL-6 production

The production of the pro-inflammatory cytokines TNF-α, IL-1β, and IL-6 in the cell cultures was detected by ELISA. The culture medium was collected and centrifuged at 12000 rpm for 10 min. The supernatants were collected and used to detect TNF-α, IL-1β, and IL-6 levels with ELISA kits, according to the manufacturer’s protocol.

### The transcription levels of TNF-α, IL-1β, IL-6, and inducible NO synthase

Total RNA was extracted using TRIzol reagent (Takara, Dalian, China) according to the manufacturer’s protocol. cDNA synthesis was performed using a reverse transcription kit (Takara, Dalian, China) according to the manufacturer’s instructions. For quantitative PCR, the cDNA was mixed with 12.5 μl of 2× SYBR Green Master Mix (BioEasy SYBR Green Real-Time PCR Kit, Bioer), 1 μl of primers (400 nM), and 9.5 μl of RNase-free water. The nucleotide sequences of the primers are shown in Table 1. Fluorescence-based quantitative PCR was performed using SYBR Premix II in an Applied Biosystems VR7500 Fast Dx Real-Time PCR Instrument. The 2^ΔΔ*C*^_T_ method was used to calculate relative quantities, as described previously, and *β-actin* was used as the internal reference gene.

**Table 1:** DNA Sequences of the primers used for real-time PCR

Gene	Primer	Sequence (5′–3′)
*TNF-α*	Forward	CAGGAGGGAGAACAGAAACTCCA
	Reverse	CCTGGTTGGCTGCTTGCTTGAAAGC
*IL-1β*	Forward	TCCAGGATGAGGACATGAGCAC
	Reverse	GAACGTCACACACCAGCAGGTTA
*IL-6*	Forward	GCCAGAGTCCTTCAGAGAGA
	Reverse	GGTCTTGGTCCTTAGCCACT
*iNOS*	Forward	TAGGCAGAGATTGGAGGCCTTG
	Reverse	GGGTTGTTGCTGAACTTCCAGTC
*COX-2*	Forward	CAGGCTGAACTTCGAAACA
	Reverse	GCTCACGAGGCCACTGATACCTA
*β-actin*	Forward	GCCACCAGTTCGCCATGGAT
	Reverse	GCTTTGCACATGCCGGAGC

### ROS levels

ROS levels were monitored using a 2′,7′-dichlorodihydrofluorescein diacetate (DCFH-DA) cell-permeant probe [21]. In this method, DCFH-DA is hydrolyzed to generate nonfluorescent membrane-permeable DCFH, which is converted into impermeable fluorogenic dichlorofluorescein (DCF) under oxidative conditions. Briefly, the cells from different groups were collected. After plating in 24-well plates at a density of 60000 cells/well, the cells were washed twice and incubated with 10 μmol/l DCFH-DA at 37°C in 5% CO_2_ for 30 min and then washed with serum-free medium to remove the extracellular DCFH-DA. Fluorescence was then determined at a 488-nm excitation wavelength and a 525-nm emission wavelength.

### Flow cytometric analysis of apoptosis

The cells were treated with hydrocortisone and FM0807 at the indicated concentrations for 20 h in serum-free medium, prior to the addition of LPS (0.5 μg/ml) for 4 h. The cells were then collected and stained with Annexin V and propidium iodide (PI) using Apoptosis Detection Kit for flow cytometric analysis. The flow cytometric analysis was performed on a BD FACSCanto II, and the data were analyzed with FlowJo 8.8.6 software (Tree Star Inc., Ashland, OR, U.S.A.). All experiments were performed with biological triplicates, and the data are representative of at least three independent experiments.

### Western blotting

The cells were preincubated with or without the indicated concentrations of FM0807 and hydrocortisone for 20 h prior to exposure to LPS (0.5 μg/ml). After 4 h, the cells were harvested. The cells were washed with PBS and treated with trypsin-EDTA. The cell pellets were obtained by centrifugation at 3000 rpm for 5 min at 4°C, lysed in lysis buffer, and centrifuged at 12000 rpm for 5 min at 4°C to obtain whole-cell lysates. Protein concentrations were determined using a BCA protein assay kit (Beyotime, China). The proteins were electroblotted on to PVDF membranes after separation via SDS/PAGE (12% gel). The transferred membranes were blocked with TBS containing 5% nonfat dried milk and 0.1% Tween-20 at 4°C for 2 h. After blocking, the membranes were incubated with primary antibodies (1:1000) overnight at 4°C with gentle shaking. Then, the blots were washed three times with Tween-20/TBS and incubated with a horseradish peroxidase (HRP)-conjugated anti-rabbit IgG secondary antibody (1:1000) for 2 h at room temperature with gentle shaking. After the membranes were washed three times for 10 min in TBS containing 0.1% Tween-20, the bands were detected using ECL Western blotting detection reagents according to the manufacturer’s protocol. β-actin was used as the loading control. The band density was measured using ImageJ software.

### Statistical analysis

All values are expressed as the mean ± S.D. Two groups were compared using an unpaired two-tailed *t* test. For multiple groups, the data were subjected to ANOVA. All experiments were repeated three times with similar results. The obtained data were statistically analyzed with SPSS 15.0 software. *P*-values less than 0.05 were considered statistically significant, and *P*-values less than 0.01 were considered notably statistically significant.

## Results

### The effect of FM0807 on the viability of RAW 264.7 cells

The effect of FM0807 on the viability of RAW 264.7 cells was assessed using an MTT assay. The results are presented in Figure 1D. FM0807, at a given concentration, had no significant effect on the cellular activity of RAW 264.7 cells compared with that of the control group. This finding demonstrated that FM0807 had no cytotoxic effects on RAW 264.7 cells.

### The effects of FM0807 on cytokine production by LPS-induced RAW 264.7 cells

As shown in Figure 1E–H, after LPS induction for 4 h, the TNF-α, IL-1β, IL-6, and NO levels significantly increased compared with those of the control group (*P*<0.01). FM0807 significantly reversed the LPS-induced TNF-α, IL-1β, IL-6 and NO levels, with *P*<0.05 or *P*<0.01 for the 10, 20, and 40 μM treatments.

### The relative transcription levels of TNF-α, IL-6, IL-1β, and iNOS

**Figure 2:**
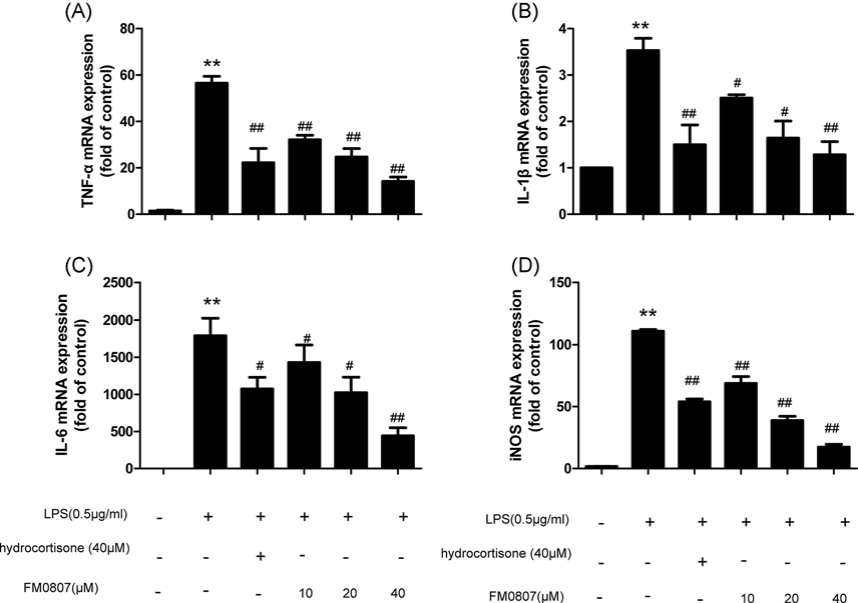
Effect of pretreatment with FM0807 on TNF-α, IL-6, IL-1β, and iNOS gene expression in LPS-induced RAW 264.7 cells (**A**) The expression of *TNF-α* mRNA. (**B**) The expression of *IL-1β* mRNA. (**C**) The expression of *IL-6* mRNA. (**D**) The expression of *iNOS* mRNA. RAW264.7 cells were pretreated with FM0807 (10, 20, 40 μM) and hydrocortisone, respectively, for 20 h prior to LPS stimulation. After 4 h of LPS stimulation, the TNF-α, IL-6, IL-1β, and iNOS mRNA levels were accessed by quantitative-RT-PCR. Data are presented as mean ± S.D. Representation of results obtained from three independent experiments are shown. **P<0.01 compared with the control group; ^#^*P*<0.01 and ^##^*P*<0.05 compared with the LPS-treated group. Abbreviation: RT-PCR, real-time PCR.

Compared with those of the control group, the transcription levels of *TNF-α, IL-6, IL-1β*, and *iNOS* mRNA were significantly increased (*P*<0.01) in the LPS-treated group. While, the FM0807 groups exhibited significantly inhibited *TNF-α, IL-6, IL-1β*, and *iNOS* mRNA transcription (*P*<0.01 or *P*<0.05) (Figure 2), compared with the LPS-treated group,. Additionally, the reduction in TNF-α, IL-6, IL-1β, and iNOS transcription occurred in a dose-dependent manner after FM0807 treatment.

### The effect of FM0807 on apoptosis in RAW 264.7 cells

**Figure 3:**
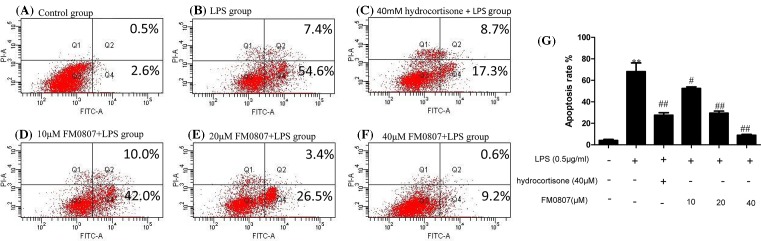
Effect of pretreatment with FM0807 on apoptosis in LPS-induced RAW 264.7 cells Flow cytometry results are represented as (**A**) cells without stimulation, (**B**) cells treated with LPS for 4 h, (**C**) cells pretreated with hydrocortisone for 20 h, (**D**–**F**) cells pretreated with FM0807 (10, 20, 40 μM), respectively, for 20 h, (**G**) bar graph representation of apoptosis rate in each group previously described. After 4 h of LPS stimulation, apoptosis rate was detected with Annexin-V and PI for flow cytometry analysis. Data are presented as mean ± S.D. Representation of results obtained from three independent experiments are shown. **P<0.01 compared with the control group; ^#^*P*<0.01 and ^##^*P*<0.05 compared with the LPS-treated group.

The antiapoptotic effect of FM0807 was investigated by flow cytometry (Figure 3). Compared with the control group, the apoptosis rate was significantly increased in the LPS-treated group (*P*<0.01). In contrast, treatment of the cells with different concentrations of FM0807 reduced the percentage of apoptotic cells (*P*<0.01 or *P*<0.05).

### The effect of FM0807 on ROS release by LPS-induced RAW 264.7 cells

**Figure 4:**
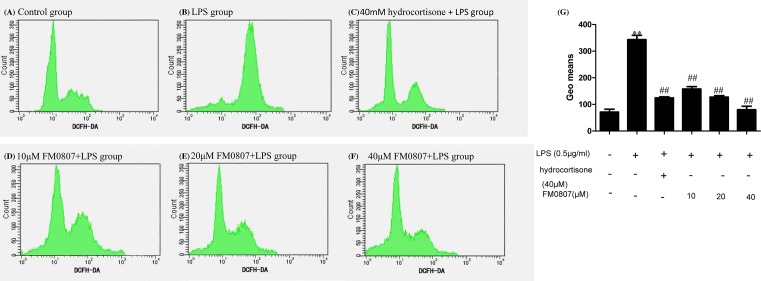
Effect of pretreatment with FM0807 on levels of ROS DCFH-DA fluorescence was confirmed at 488 nm excitation and 525 nm emission. (**A**) Cells without stimulation, (**B**) cells treated with LPS for 4 h, (**C**) cells pretreated with hydrocortisone for 20 h, (**D**–**F**) cells pretreated with FM0807 (10, 20, 40 μM) respectively for 20 h, (**G**) bar graph representation of geo means in each group previously described. **P<0.01 compared with the control group; ^##^*P*<0.01 compared with the LPS-treated group.

Previous studies have indicated that ROS are involved in sepsis. To determine whether FM0807 can inhibit ROS induced by LPS, RAW 264.7 cells were treated with the DCFH-DA dye after treatment with different concentrations of FM0807. The DCFH-DA fluorescence results are shown in Figure 4. The control group and the LPS-treated group had obviously different levels of fluorescence (*P*<0.01), suggesting that LPS can stimulate the cells to produce ROS. After pretreatment with FM0807, the DCFH-DA fluorescence in the LPS-induced cells was reduced (*P*<0.01). These results suggest that FM0807 had an inhibitory effect on the level of ROS in LPS-treated cells.

### The effect of FM0807 on the apoptosis pathway after stimulation with LPS

**Figure 5:**
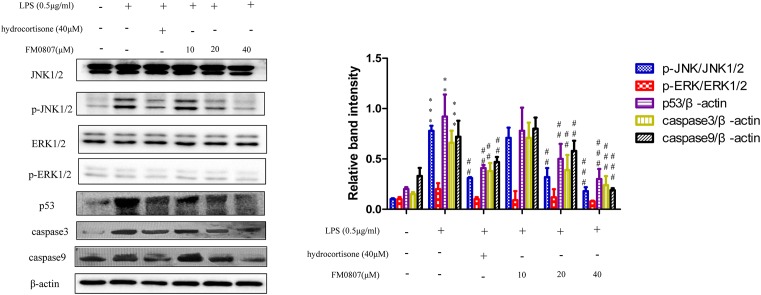
Inhibitory effects of FM0807 on the LPS-induced apoptosis signaling pathway (**A**) Representative Western botting analysis of p-JNK/JNK1/2, p-ERK/ERK1/2, p53, caspase-3, and caspase-9 with β-actin protein expression as an internal control. (**B**) Quantitation of Western blot analysis of p-JNK/JNK1/2, p-ERK/ERK1/2, p53, caspase-3, and caspase-9. **P<0.01 and ***P<0.005 compared with the control group; ^##^*P*<0.05 and ^###^*P*<0.0001 compared with the LPS-treated group.

To further investigate the molecular mechanism of the anti-inflammatory effect of FM0807, the effects of different concentrations of FM0807 on the apoptosis signaling pathway after stimulation with LPS were observed. We explored the expression of p-JNK/JNK1/2 and p-ERK/ERK1/2 in the MAPK signaling pathway. p53 is activated in response to ROS, resulting in the activation of caspase-3 and caspase-3. Hence, p53, caspase-3 and caspase-9 were also detected. The results are shown in Figure 5. p-JNK/JNK1/2, p53, caspase-9, and caspase-3 levels increased after LPS stimulation. p-ERK/ERK1/2 exhibited no changes in the present study. The changes in p-JNK/JNK1/2, p53, caspase-3, and caspase-9 were reversed by treatment with FM0807 at doses of 20 and 40 μM.

## Discussion

Sepsis is characterized as a disorder of inflammatory cytokine and ROS production. Antioxidants can inhibit or alleviate the adverse effects of free radicals on the body, playing a significant role in the prevention and treatment of sepsis [22–24]. Curcumin has a wide range of pharmacological effects, such as antitumor, anti-inflammatory, and hypolipidemic effects [16,25–27]. Recent evidence has demonstrated that curcumin can alleviate LPS-induced sepsis by suppressing oxidative stress-related inflammation [16]. The curcumin derivative FM0807 was synthesized in our laboratory, where its antioxidant and anti-inflammatory activities were evaluated. Our study focussed on the inhibition of inflammatory cytokines to explore the antioxidant and anti-inflammatory effects of FM0807 *in vitro*. We found that pretreatment with FM0807 inhibited the inflammatory factor TNF-α, IL-1β, IL-6, iNOS at the protein and gene levels. FM0807 also inhibited ROS and apoptosis. We further demonstrated that the activation of the ROS/JNK/p53 signaling pathway was inhibited by FM0807 in RAW 264.7 cells. Collectively, our data demonstrated that FM0807 has anti-inflammatory activity *in vitro*. The anti-inflammatory activity of FM0807 may be mediated by the ROS/JNK/p53 signaling pathway.

As one of the inflammatory mediators involved in sepsis, TNF-α is an important inflammatory cytokine during the early stage of inflammation and is mainly produced by monocytes and macrophages. TNF-α can activate the cytokine cascade, resulting in a ‘waterfall effect’ and triggering the synthesis of secondary inflammatory mediators, such as IL-1β, IL-6, and NO [28]. The excessive production of TNF-α, IL-1β, IL-6, and other cytokines can further activate macrophages. Then, the cells will induce the expression of iNOS, which can produce and release a large amount of NO. NO is an effector molecule by which activated macrophages and tumor cells kill pathogenic microorganisms. NO is also an important mediator of inflammation. The excessive production of NO plays a variety of roles in the development of inflammation. Cytokines also play an important role in the development of sepsis. In summary, TNF-α, IL-1β, IL-6, and NO are important indicators for evaluating inflammatory responses and therapeutic effects in clinical and basic research. The inhibition of inflammatory cytokines is a target for sepsis therapy. Gupta et al. showed that curcumin can inhibit the production of inflammatory cytokines and NO [29–31]. In this study, an *in vitro* inflammation model was established successfully. RAW 264.7 cells stimulated with LPS release TNF-α, IL-1β, IL-6, and NO. In this context, we found that the curcumin derivative FM0807 has inhibitory effects on TNF-α, IL-1β, IL-6, and NO at the protein and gene levels in LPS-induced RAW 264.7 cells, indicating that FM0807 has anti-inflammatory activity.

ROS are highly reactive and include superoxide anion radicals, hydroxyl radicals, and hydrogen peroxide. The mitochondrial respiratory chain is a major source of ROS. Under normal circumstances, the production and elimination of free radicals *in vivo* is balanced. When free radical production becomes excessive or the antioxidant system fails, the metabolism of free radicals in the body becomes unbalanced, leading to cellular damage, this process has a relationship with sepsis [32,33]. In this study, we found that FM0807 inhibited LPS-induced cells production of ROS in LPS-induced RAW 264.7 cells. ROS and TNF-α contribute to apoptosis, and apoptosis is a key process in sepsis. Apoptosis is characterized by the activation of caspases. Previous studies have indicated that mitochondria play an important role in apoptosis, which activates caspase-9 and caspase-3. We explored the effect of FM0807 on apoptosis in LPS-induced RAW 264.7 cells. Our study suggested that FM0807 has inhibitory effects on the apoptosis rate.

Furthermore, the mechanism of this inhibitory effect was investigated in the present study. The MAPK family participates in many cellular processes, such as cellular differentiation, proliferation, and apoptosis. The role of MAPKs in apoptosis has been well established in a variety of models, including mitochondrial dysfunction and oxidative stress. A previous study found that ROS accumulation led to the activation of JNK and resulted in apoptosis [34,35]. The activation of ERK is usually involved in cell survival and proliferation, while JNK and p38 are implicated in the promotion of apoptosis by activating a series of genes [36,37]. p53, which can be activated by JNK, acts as a key player in apoptosis. p53 can activate caspase-9 and contribute to the activation of caspase-3. We investigated the influence of FM0807 on the JNK/p53 signaling pathway in LPS-induced RAW 264.7 cells. Our results indicated that FM0807 inhibits JNK/p-JNK but has no effect on ERK/p-ERK. JNK can activate p53, which activates caspase-9, contributing to the activation of caspase-3. Our study proved that FM0807 can decrease p53 levels, resulting in the reduction in caspase-9 and caspase-3. The curcumin analogs, displaying the LPS-induced inflammation inhibition, have been previously reported to participate in the Toll-like receptor (TLR) signaling complex through the myeloid differentiation protein 2 (MD2) and the TLR4 [38,39]. Here, we concluded that ROS accumulation and the subsequent activation of the JNK/p53 signaling pathway play vital roles in LPS-induced apoptosis.

Our present findings suggest that the curcumin derivative FM0807 can inhibit pro-inflammatory mediators, apoptosis, and ROS. These results indicate that FM0807 has antioxidant and anti-inflammatory activities *in vitro*. The anti-inflammatory activity of FM0807 may be mediated by the JNK/p53 signaling pathway. FM0807 may be a promising candidate for a novel inhibitor of inflammation in sepsis. However, further studies are needed to demonstrate how FM0807 targets and affects p53.

## Abbreviations

DCF: dichlorofluorescein
DCFH-DA: 2′,7′-dichlorodihydrofluorescein diacetate
DMEM: Dulbecco’s modified Eagle’s medium
ERK: extracellular signal-regulated kinase
HRP: horseradish peroxidase
IL: interleukin
iNOS: inducible nitric oxide synthase
JNK: c-jun NH_2_-terminal kinase
LPS: lipopolysaccharide
MAPK: mitogen-activated protein kinase
NO: nitric oxide
PI: propidium iodide
RNS: reactive nitrogen species
ROS: reactive oxygen species
TLR: Toll-like receptor
TNF-α: tumor necrosis factor-α
